# Performance Evaluation of Radiation-Shielding Materials and Process Technology for Manufacturing Skin Protection Cream

**DOI:** 10.3390/ma16083059

**Published:** 2023-04-12

**Authors:** Seon-Chil Kim

**Affiliations:** Department of Biotechnology, Keimyung University, 1095 Dalgubeol-Daero, Daegu 42601, Republic of Korea; chil@kmu.ac.kr; Tel.: +82-10-4803-7773

**Keywords:** medical radiation, barium sulfate, bismuth oxide, protective cream, shielding

## Abstract

Personnel using X-ray devices, the main source of radiation in medical institutions, are primarily affected by scattered rays. When interventionists use radiation for examinations/treatments, their hands may enter the radiation-generating area. The shielding gloves used for protection against these rays restrict movement and cause discomfort. Here, a shielding cream that directly adheres to the skin was developed and examined as a personal protective device; further, its shielding performance was verified. Bismuth oxide and barium sulfate were selected as shielding materials and comparatively evaluated in terms of thickness, concentration, and energy. With increasing wt% of the shielding material, the protective cream became thicker, resulting in improved protection. Furthermore, the shielding performance improved with increasing mixing temperature. Because the shielding cream is applied to the skin and has a protective effect, it must be stable on the skin and easy to remove. During manufacturing, the bubbles were removed, and the dispersion improved by 5% with increasing stirring speed. During mixing, the temperature increased as the shielding performance increased by 5% in the low-energy region. In terms of the shielding performance, bismuth oxide was superior to barium sulfate by approximately 10%. This study is expected to facilitate the mass production of cream in the future.

## 1. Introduction

As the amount of radiation used for diagnosis and treatment in medical institutions increases, the scope and frequency of operators and patients being exposed to radiation increase [1]. Medical radiation accounts for approximately 15% of the radiation that humans are exposed to in the natural state, and the radiation exposure of medical workers and patients has recently increased owing to the increase in radiation examinations in hospitals, interventional procedures, and CT examinations [2,3]. Medical personnel wear special aprons as shielding suits to protect against X-rays, which are mainly used in hospitals. However, in reality, these aprons are limited in protecting all parts of the human body [4,5]. In particular, imaging operators are virtually defenseless against radiation exposure because they rely on manual techniques and tactile sensations of the hand [6].

To manufacture clothing that protects against medical radiation, the shielding fabric material needs to be light-weight, thin, and flexible. To satisfy these demands, the development and discovery of shielding materials for various materials are important [7,8]. Furthermore, efforts must be made to shield personnel against not only direct radiation but also low-dose indirect radiation, including scattered radiation. Low-dose radiation refers to radiation with a dose of 100 mSv or less [9]. According to BEIR VII, low-dose radiation is explained by a linear proportionality theory without a threshold and is said to have the potential to cause cancer and genetic disorders [10]. The ICRP Recommendation (ICRP Publ. 60) proposed a dose limit of 500 mSv/year for equivalent skin dose and equivalent dose to the hands and feet for workers to prevent deterministic effects [11].

Therefore, reducing the exposure of patients and medical workers requires shielding a large area. In this study, we designed a material that could temporarily and simply protect the skin of medical personnel from scattered rays in areas uncovered by the apron. We report the results of research on the development of new materials and the improvement of shielding performance. In previous studies, the development of shielding materials in the form of sheets, fibers, and films that could be used in medical institutions was studied through the discovery of eco-friendly shielding materials. However, there are material limitations in achieving the required thinness or light weight characteristic of the shield [12,13]. Therefore, new material shielding technology with very low wearing comfort and resistance is required, as well as a creative approach for this purpose. In this study, a cream material that can be used as a shielding material directly on the skin was proposed. Furthermore, we intend to expand the user’s shielding area and the selection range of personal protective equipment for medical radiation [14]. Hands are one of the areas where doctors are most exposed to radiation in hospitals; hence, there are products such as shielding gloves. These products are suggested for sensory activities [15]. However, they restrict movement and sometimes cause discomfort. In this study, a radiation-protection cream, which is a product that can be easily applied to the skin of the hand when necessary, is manufactured, and the shielding characteristics are analyzed.

Existing shielding creams do not present a quantitative shielding effect according to the manufacturing method, and they mainly suggest functional effects. Reproducibility of the shielding rate is difficult, and it depends on the manufacturing method of the shielding cream [16]. Hence, in this study, the shielding performance of the cream, using eco-friendly radiation-shielding materials that are harmless to the human body, was evaluated. The environmentally friendly materials used were barium sulfate and bismuth oxide. Most existing skin protectants require a formulation technology with excellent stability and percutaneous absorption [17]. However, regarding protective cream, because it is not a skin-friendly material (such as absorption), there is a formulation technology that can safely protect radiation from the outside of the skin and be easily removed. Therefore, it may be advantageous to demonstrate the effect of scattering low-dose radiation outside the skin rather than absorbing radiation [18].

The technology to block UV rays on the skin uses a scattering agent. However, regarding high-intensity incident energy, the effect of protecting the material by evenly dispersing the radiation shielding effect on the transdermal surface must be explained [19]. Therefore, barium sulfate and bismuth oxide must be formulated to ensure that they are evenly dispersed on the skin, such as by a shielding sheet or shielding film manufacturing process [20].

As this dispersion technology corresponds to the most important mixing condition in process technology, this study also intends to perform a comparative evaluation of the shielding performance of the mixing method in the cream-manufacturing mixing condition. In this study, the characteristics of a protective cream made of barium sulfate and bismuth oxide, which are shielding materials, were compared and evaluated, and the best mixing conditions were proposed by considering the shielding effect according to the wt% of the shielding material. The possibility of commercializing this shielding cream for imaging procedures was also verified by comparing the shielding effect of existing surgical shielding gloves. Through these studies in the future, we intend to present a quantitative index direction for process technology for mass producing various skin coating materials that can be directly shielded from the outside of the skin.

## 2. Materials and Methods

Barium sulfate is an environmentally friendly radiation-shielding material that has a density of 4.5 g/cm^3^, while bismuth oxide has a density of 8.9 g/cm^3^. Furthermore, it was selected as a cream-type formulation material that can be used for the outer skin because of its excellent affinity with other additives, such as emulsifiers [21]. First, this study focused on the properties of protective creams that make them different from existing cosmetics. (1) This cream is not to be absorbed into the skin; (2) It must have the property of being easily removed after being dispersed on the skin and evenly coating the skin surface. Scattered rays generated by X-rays irradiated from the outside can be understood as low-dose radiation generated from the cross-sectional area of the surface. The incident i, j for the total area $P\left(\gamma \right)$ is the correlation between the electron density and area. Equation (1) can be explained by [22]—the scattering area (r) must be shielded.

(1) $$P\left(\gamma \right)=4\pi \gamma^{2}\sum_{i,j\neq 1}\rho ij\left(\gamma \right)$$

The area to be shielded is attenuated with respect to the thickness $\left(x\right)$ of the material through which the intensity of the incident energy is transmitted [23]. Therefore, in the case of X-rays incident on the diagnostic area, the intensity is weakened, as shown in Equation (2) because of the interaction with the shielding material while passing through the shielding body [24].

(2) $$I=I_{0e}{-}^{ux},$$

where I is the X-ray intensity after penetrating the shield, *I*_0_ is the incident X-ray intensity, µ is the linear damping constant (cm^−1^), and X is the thickness of the shield (cm).

Therefore, in this study, we developed a cream-type material that can be applied to a large area and attempted to track the change in shielding rate according to thickness. Barium sulfate and bismuth oxide were compared for the materials of the prepared protective cream, and the shielding performance was further evaluated using wt%, the mass of the solute of the shielding material.

The powder particles of the shielding material used in the manufacture of the protective cream (purity of 99.0% or more and 4 μm or less) were not resistant to contact with the skin, and the minimum processing size was selected to reduce voids. Barium sulfate and bismuth oxide are eco-friendly shielding materials that are manufactured and used in film, sheet, and fiber forms. Compared to lead, they are non-toxic to the human body and lighter than tungsten. Therefore, eco-friendly shielding materials were selected for future use. The manufacture of protective creams applies a method of mixing shield materials with existing creams and includes lubricants, wetting agents, surfactants such as glycerin, and emulsifiers such as glyceryl stearate in aqueous organic carriers, which are raw materials for creams. The barium sulfate was blended in 5 wt% units from 10 to 35 wt%.

The criterion for blending is viscosity, and the maximum mixing ratio that can be applied to the skin is 35 wt%. Furthermore, it is difficult for the user to apply it to the skin if it is abnormal. Therefore, in this experiment, the mixing ratio was the same, and the shielding material was slowly added to the aqueous organic carrier during the mixing process and mixed at a speed of 3000 rpm with a homogen mixer (Primix, Homo Mixer, Tokyo, Japan) at a temperature of 70 °C for 8 min. The temperature and speed of the homo mixer were changed and compared to determine the cause of the improved dispersion of the shielding material.

The temperature was evaluated based on the low temperature (50 °C) and high temperature (70 °C), and the mixing speed was compared for 8 min at 1000, 2000, and 3000 rpm. For comparison, magnified images were analyzed with the naked eye using an optical microscope (FESEM; field emission scanning electron microscope, Hitachi, S-4800, Tokyo, Japan).

**Figure 1:**
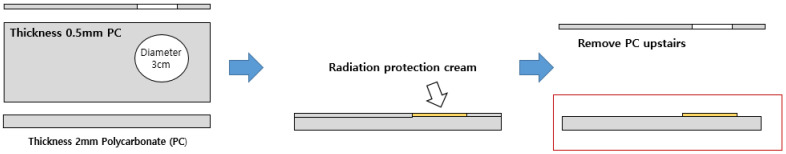
Thickness control plate of protective cream to evaluate shielding performance.

The shielding performance was compared and analyzed by the shielding rate using seven plates, as shown in Figure 1 [25]. The thickness was compared and measured for five thicknesses ranging from 0.5 to 3 mm. The selected standard thickness was calculated as the thickness that can be toted on the skin and worn with sterile surgical gloves.

Using the diagnostic X-ray (MOBIX-1000, Listem, Inchun-City, Republic of Korea, 2010), the experiment was conducted based on tube voltages of 40, 60, 80, 100, and 120 kVp.

**Figure 2:**
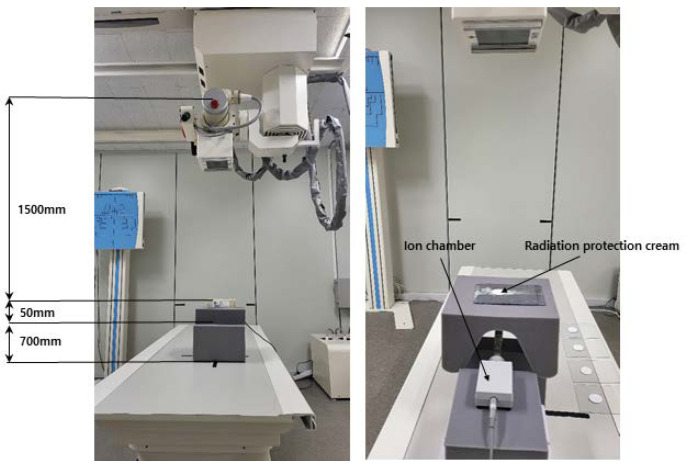
X-ray shielding performance evaluation method.

The radiation detection dosimeter was constructed and tested, as shown in Figure 2, using an ion chamber ionizer (Radical 9015 with a 6 cc ion chamber, Radcal Co., 2017, Correction. 2020, Monrovia, CA, USA) [26].

The experimental results were compared and evaluated for shielding performance with the shielding ratio calculated as shown in Equation (3) [27].

(3) $$\text{X-ray}\;\mathrm{shielding}\;\mathrm{rate}(\%)\;\left(1-\frac{k}{k_{0}}\right)\times 100$$

where *k*_0_ is the incident dose (mR) and *k* is the transmitted dose (mR).

## 3. Results

**Figure 3:**
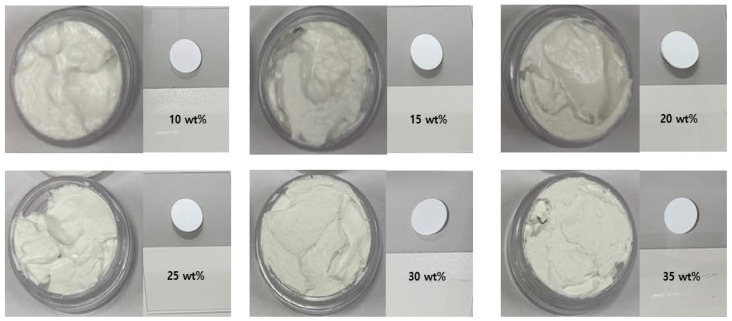
Barium sulfate protective cream according to wt% observed with the naked eye.

**Figure 4:**
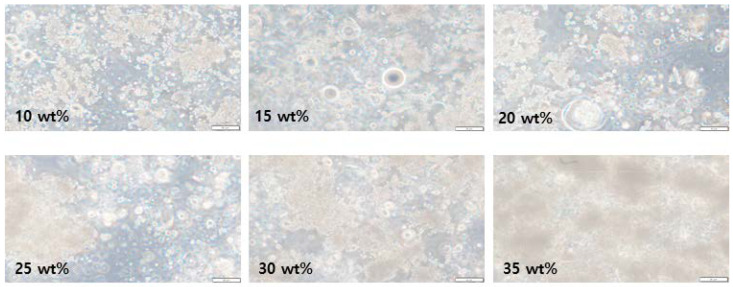
Degree of dispersion of barium sulfate according to wt% observed with an optical microscope (×100).

The protective cream was manufactured using two types of barium sulfate and bismuth oxide, and the shielding performance was compared and analyzed based on barium sulfate, which is easy to remove after use. We compared lead surgical shielding gloves (Genova, 2019, Italy) currently used in interventional procedures at medical institutions [28]. Figure 3 shows the appearance of the protective cream applied to the plate, and Figure 4 shows an image of the particle distribution of the shielding material according to the wt% through an optical microscope. To the naked eye, the shapes of the creams look identical. However, the dispersion of the shielding material according to the wt% can be distinguished using a microscope image. Furthermore, the higher the wt%, the better the dispersion. As shown in Figure 4, the area of the barium sulfate contained in the aqueous organic carrier becomes more visible as the wt% increases, which can be attributed to the excellent dispersion. The degree of thick overlapping is also more visible as the wt% increases, which is essential for improving the shielding performance.

Barium sulfate and bismuth oxide were compared as shielding materials for radiation-protection creams. The particle sizes were identical. Their shielding performances in cream form are shown in Table 1. The mixed concentration of the shielded material was evaluated at 20 wt%, and bismuth oxide was approximately 10% higher than barium sulfate. There was no significant difference when the thickness was low. However, a difference in the shielding performance was observed when the thickness was high. There was no significant difference considering only the shielding performance, and in particular, bismuth oxide required a little more time than barium sulfate to be removed from the skin.

**Figure 5:**
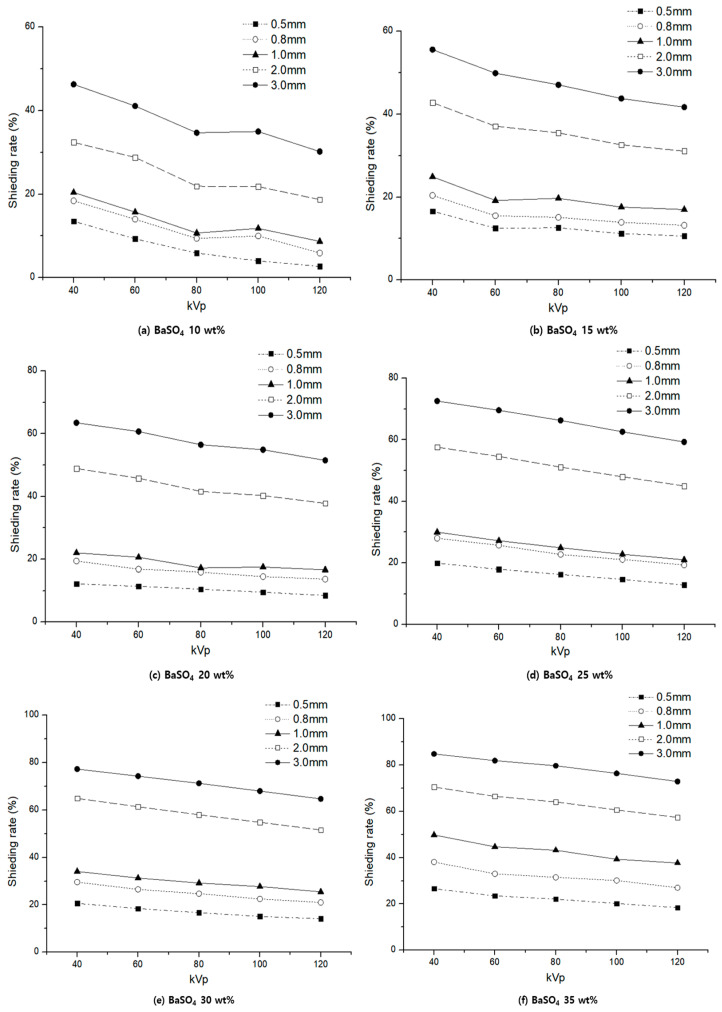
Comparison of shielding rate by wt% by thickness (barium sulfate).

The shielding performance for each thickness of the protective cream made of barium sulfate shielding material was analyzed, as shown in Figure 5. The thickness of the protective cream applied to the skin is an important parameter.

From the graph, when comparing the shielding performance by incident energy and thickness, a difference is shown in the low-energy region. However, there is no significant difference in the region with high tube voltage, which is the region with high energy strength. In addition, the higher the wt% of the shielding material in the protective cream, the better the shielding effect.

**Figure 6:**
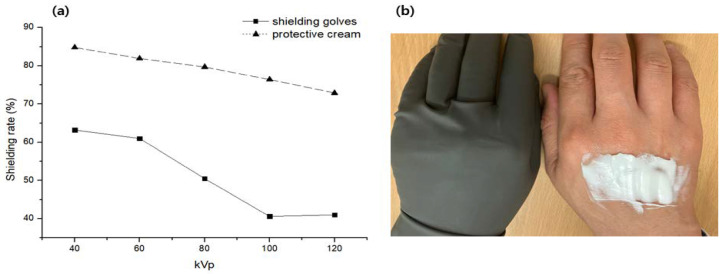
Comparison of shielding gloves and protective cream (30 wt%); (**a**) tungsten-based surgical shielding gloves (0.45 mm), and (**b**) barium sulfate-based protective cream (3.0 mm).

Currently, surgical shielding gloves are commonly used and have a thickness of 0.45 mm. They are molded by mixing tungsten powder (4 μm) with polyethylene polymer material and have excellent flexibility. Shielding gloves and protective cream (30 wt%) with a thickness of 3.0 mm were compared, as shown in Figure 6. Despite the difference in thickness, the protective cream is more effective than shielding gloves. However, when applied to a thickness of 3.0 mm, as shown in the results, the skin may become very rigid. Therefore, a thickness of approximately 3.0 mm, which is not a significant problem for wearing general surgical gloves with shielding cream, was found.

**Figure 7:**
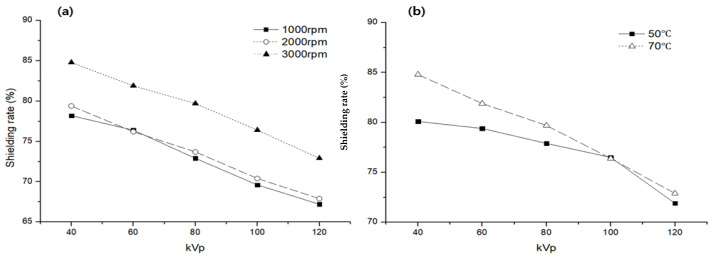
Changes in the shielding performance of the protective cream according to the speed (rpm) and temperature of the homo mixer: (**a**) shielding performance according to rpm, (**b**) shielding performance according to temperature.

**Figure 8:**
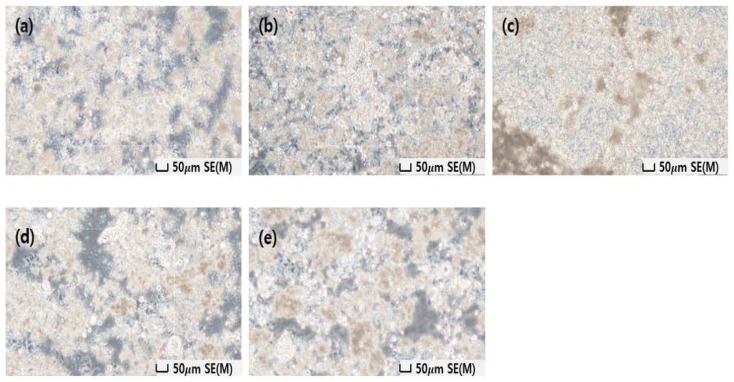
Degree of dispersion of the protective cream according to the speed (rpm) and temperature of the mixer observed with an optical microscope (×40); (**a**) 1000 rpm, (**b**) 2000 rpm, (**c**) 3000 rpm, (**d**) 50 °C, (**e**) 70 °C.

To review the process technology to increase the shielding performance in the manufacturing process of the protective cream, the shielding performance and dispersion of the cream were reviewed according to the temperature and speed (rpm) of the homo mixer during the mixing process. The results are shown in Figure 7. In the process of mixing the water-based organic carrier and the shielding material, air bubbles are removed, which is an important process that has a better effect as the rotation speed of the mixer increases because it affects the shielding performance. Furthermore, the temperature is not significantly affected by low or high temperatures. However, in low-dose shielding, a high temperature is effective, indicating a slight difference. Figure 8 shows an enlarged image of the smear, and it can be interpreted that there is a difference in the dispersion of the shielding material according to the mixing method. A comparison of Figure 8d,e reveals that Figure 8e has better dispersion.

**Figure 9:**
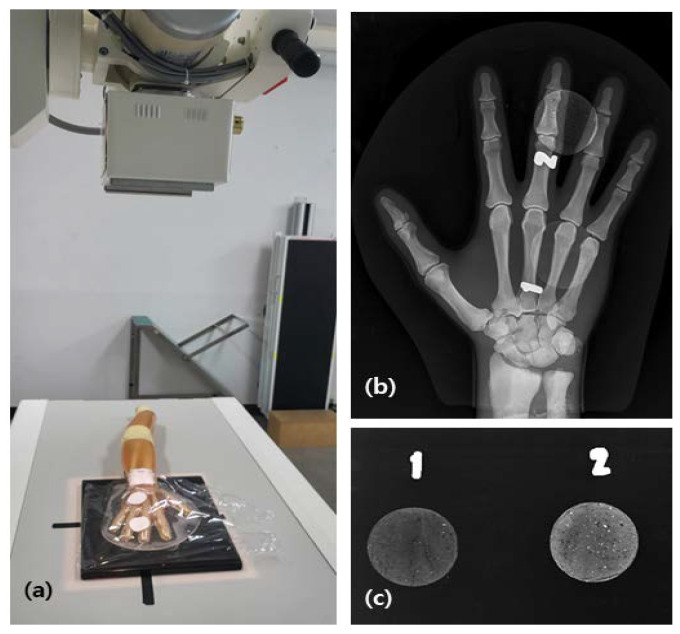
Radiation Imaging Analysis Using Human Hand Phantom: (**a**) filming scene to obtain an X-ray image of protective cream; (**b**) image of 1.0 mm protective cream on a phantom; (**c**) image showing different concentrations of 0.1 mm barium sulfate (1: 20 wt%, 2: 30 wt%).

When applied on the last hand, the concentration analysis of the X-ray image observed in the duct is shown in Figure 9. As shown in the image, there was a difference in the X-ray image density according to the wt%.

## 4. Discussion

The purpose of low-dose radiation shielding in medical institutions is to minimize the disadvantages of radiation [29]. A large number of X-rays are used in medical practice. However, efforts have been made to defend through distance, time, and shielding [30]. Most low-dose radiation in medical institutions is scattered rays, and efforts to predict and defend against them must precede them. Simultaneously, a shielding body capable of active shielding is required. Shielding materials used in medical institutions include those used in April and gloves. Most shielding fabrics are materials that mix shielding materials with polymeric materials [31]. Furthermore, the flexibility of the material is secured, but the proximity to the body is lowered, and the accessibility of the shield is lowered owing to the weight. In addition, from the user’s perspective, the focus is on the direction and distance of the radiation source rather than the shielding body [32]. Therefore, a lightweight material that is thin and has excellent flexibility to easily remove scattered rays from around the body must be used [33]. However, even if the condition is satisfied owing to the various postures of the operator or the patient, there will be difficulties in accessibility owing to individual differences. In this study, we investigated a material that can be applied to the skin in the form of a lotion or cream to shield against a certain amount of indirect radiation, such as a sunscreen that blocks ultraviolet rays.

The shielding cream differs in its performance depending on the intensity of the incident energy, and it is important to maintain a constant thickness of the cream to obtain a protective effect by dispersing the radiation energy incident on the skin. Therefore, in this experiment, the shielding effect of thickness was tracked and presented, and the focus was placed on the hand. Furthermore, the thicker the layer, the better the shielding effect. However, hardening occurred when it dried on the skin on the back of the hand. When the moisture content was increased to reduce the curing phenomenon, unevenness of the coated surface occurred owing to slipping [34,35]. To solve this problem, the wt% was increased, resulting in improved shielding performance and increased fixation. As shown in the experimental results, applying 3.0 mm to the skin is a large amount, and for current activities, approximately 1.0 mm is not too large for daily activities. To remove moisture in the future and reduce peeling, which is a powdering phenomenon, 1.0 mm is the most effective, and in the case of 3.0 mm, effective activities were possible only when external gloves were worn. Therefore, the prepared protective cream has the advantage that it can be applied to the back of the hand before putting on general surgical gloves. Therefore, the use of bismuth oxide as a shielding material is very effective. However, barium sulfate is easy to remove. If the particles of the shielding material, such as nanoparticles, are made smaller, the density increases and the pores decrease, which can be effective for shielding. Furthermore, it is difficult to remove after use, and owing to the problem of the shielding material in the protective cream being absorbed into the cells, the particle size in this study was 4 µm [36]. To reduce the gap between the shielding materials during the manufacturing process of the protective cream, the difference in the process technology of the stirring and mixing processes was also reviewed. Additionally, the process of removing air bubbles by increasing the mixing speed, without being significantly affected by the mixing speed and temperature, directly or indirectly affects shielding performance [37]. Therefore, the protective cream must also play a role in reducing the gap between the particles of the shielding material, similar to the manufacturing process of the shielding sheet and film [38]. The limitation of this study is that the shielding material of the protective cream was limited to barium sulfate and bismuth oxide. Despite protective creams being manufactured using various materials, such as boron, materials that do not cause problems with skin safety will be used preferentially, as they are applied directly to the skin [39]. The main reason for the increase in the exposure dose of interventionalists in medical institutions is an increase in the type and frequency of interventional procedures. As shown in the results of this study, a shield that can be worn continuously and at all times is required. The protective cream is considered an effective method for secondary shielding on the skin at a minimum [40,41].

## 5. Conclusions

In this study, a radiation-shielding cream was developed for low-dose radiation shielding in medical institutions, and its shielding performance was evaluated. Barium sulfate and bismuth oxide were used as shielding materials; as the wt% content increased, the difference between their shielding performances was approximately 5–10% for each energy and >5–8% depending on the thickness. During the manufacturing process, the shielding performance improved by 5% with increasing stirring speed and temperature. Further, bismuth oxide was superior to barium sulfate by approximately 10% in terms of shielding performance. The difference in shielding performance appeared as a difference in the degree of dispersion of the shielding material. Therefore, the use of medical radiation-protection cream has a shielding effect on the skin on the back of hand.

## Figures and Tables

**Table 1:** Comparison of shielding performance by energy according to shielding material characteristics.

Thick-Ness	40 kVp		60 kVp		80 kVp		100 kVp		120 kVp	
	A	B	A	B	A	B	A	B	A	B
0.5	13.7	12.2	12.6	11.4	11.1	10.5	9.9	9.5	8.6	8.5
0.8	22.0	19.4	18.0	16.8	16.5	15.9	15.1	14.5	13.9	13.7
1.0	23.9	22.0	23.1	20.6	18.3	17.2	17.9	17.5	17.4	16.6
2.0	52.4	48.9	48.8	45.8	44.2	41.6	42.1	40.3	40.1	37.8
3.0	70.1	63.5	66.9	60.7	58.4	56.5	55.4	54.9	47.4	51.5

A: Bismuth oxide (20 wt%), B: Barium sulfate (20 wt%).

## Data Availability

Not applicable.
